# The Relationship between Chocolate Consumption and the Severity of Acne Lesions−A Crossover Study

**DOI:** 10.3390/foods13131993

**Published:** 2024-06-24

**Authors:** Magdalena Daszkiewicz, Dorota Różańska, Bożena Regulska-Ilow

**Affiliations:** 1 “5D” Cosmetology and Aesthetic Medicine Clinic, 53-674 Wrocław, Poland; magdaiza.daszkiewicz@gmail.com; 2 Department of Dietetics and Bromatology, Wroclaw Medical University, 50-556 Wroclaw, Poland; bozena.regulska-ilow@umw.edu.pl

**Keywords:** acne vulgaris, chocolate, diet, acne exacerbation, crossover study

## Abstract

The aim of this study was to assess the relationship between the daily consumption of 50 g of chocolate with 85% cocoa content and the severity of acne lesions. Methods: The study involved 92 participants with acne who were divided into two groups, A (*n* = 51) and B (*n* = 41). In the first week, both groups had to follow an anti-inflammatory diet (AID), then for the next 4 weeks, group A continued on with the AID, and group B followed an AID with chocolate. After this time, group B started a 4-week AID without chocolate, and group A started a 4-week AID with chocolate. The severity of acne lesions was assessed using the Investigator’s Static Global Assessment scale, where zero points indicated no lesions and five points indicated severe acne. Results: As a result of the consumption of 50 g of chocolate, a statistically significant intensification of acne lesions was observed in both groups. After 4 weeks of following the chocolate diet, the severity of acne lesions increased from 2.5 ± 0.7 to 3.4 ± 0.8 points (*p* < 0.0001) in group A, and from 2.4 ± 0.7 to 3.5 ± 0.6 points (*p* < 0.0001) in group B. Overall, chocolate intake contributed to the exacerbation of acne lesions by one point in 65 participants, by two points in 13 participants and by three points in one participant. Conclusions: The obtained results suggest that daily consumption of 50 g of chocolate with 85% cocoa content, even with an anti-inflammatory diet, may intensify acne lesions in this study group. However, it remains unclear which chocolate components may lead to the exacerbation of acne.

## 1. Introduction

The modern understanding of the pathogenesis of acne vulgaris is constantly evolving. The authors of the studies conducted so far have shown that the pathophysiology of acne is influenced by both genetic and hormonal factors, as well as inflammatory and environmental factors. Diet is one of the factors associated with the pathology of skin lesions. The appearance of acne lesions in some populations after changing from a non-processed diet to a Western diet suggests that dietary modifications may play an important role in the pathogenesis of acne. It has been shown that the adoption of the Western diet, including processed foods, dairy products and refined sugars, by the population of Kitavan Island in Papua New Guinea and Aché foragers in Paraguay has contributed to the development of acne [1]. The authors of numerous studies have demonstrated the relationship between selected dietary ingredients and the modification of acne lesions. It has been shown, among other things, that a high glycemic index (GI) and glycemic load (GL) in the diet influence the pathways involved in the pathogenesis of acne [2]. Research conducted by Burris et al. [3] showed that an increase in GI and GL in the diet exacerbates acne. Moreover, insulinogenic amino acids contained in milk, which are responsible for the high insulin index of milk, also exacerbate acne lesions [4].

Due to the role of *Cutibacterium acnes* in the development of acne, the balance of the intestinal and skin microbiome has become a subject of research interest. A double-blind study compared the effect of using a liquid supplement containing *Lactobacillus rhamnosus GG* for 12 weeks compared to a group receiving a placebo. The results indicated a significant reduction in the number of acne lesions in participants who received the probiotic supplement [5].

With reference to elimination diets, chocolate and its effect on acne has been a common subject of research. One of the first studies on the impact of chocolate consumption on the exacerbation of acne lesions was conducted in 1969 by Fulton et al. [6] in a group of 65 respondents. It was shown that excessive chocolate consumption did not adversely affect sebum secretion and, therefore, did not exacerbate acne lesions. This study was criticized by scientists both for its failure to take into account the dietary properties of chocolate [7] and for its quantification of skin lesions [8]. However, a study conducted in 2014 showed a statistically significant intensification of acne lesions after consumption of cocoa over a period of seven days [9].

Cocoa beans, from which chocolate is made, contain many compounds, including polyphenols, which have strong antioxidant, anti-free radical, anti-inflammatory and antithrombotic properties. Although their mechanism of action has not yet been thoroughly investigated, it has so far been shown that flavonoids have antioxidant and antiplatelet effects, which contribute to the better functioning of the cardiovascular system [10]. Chocolate, with a high content of procyanidin, on the one hand, increases the concentration of prostacyclin in the blood plasma, and on the other hand, reduces the concentration of leukotrienes, thus indicating anti-inflammatory and protective properties for blood vessels [11].

In turn, clinical studies show that consuming chocolate contributes to the improvement of cognitive functions [12]. Less researched potential benefits of chocolate consumption include its anti-cancer [13] and anti-microbial properties [14]. However, cocoa consumed in the form of high-energy chocolate may have potentially harmful effects when consumed excessively, which include, among others, the risk of weight gain [15].

The aim of this study was to assess the relationship between the daily consumption of 50 g of chocolate with 85% cocoa content and the severity of acne lesions.

## 2. Materials and Methods

The crossover study involved 92 people (88 women and 4 men). The participants of the study were patients of the “5D Clinic” Cosmetology and Aesthetic Medicine Clinic in Wrocław, Katowice, Kraków and Łódź. Informed consent was obtained from all subjects involved in the study. The study group included people aged 18 to 45.

The inclusion criteria for the study were as follows:

(a) occurrence of acne lesions,
(b) no antibiotic and steroid treatment 6 months before examination or supplement use,
(c) no treatment with retinoids 6 months before examination,
(d) age over 18,
(e) no comorbidities.

The severity of acne lesions was determined based on the Investigator’s Static Global Assessment (ISGA), where 0 points indicate no lesions and 5 points indicate severe acne [16]. The research was approved by the bioethics committee (No. KB-821/2020).

### 2.1. Study Design

**Figure 1:**
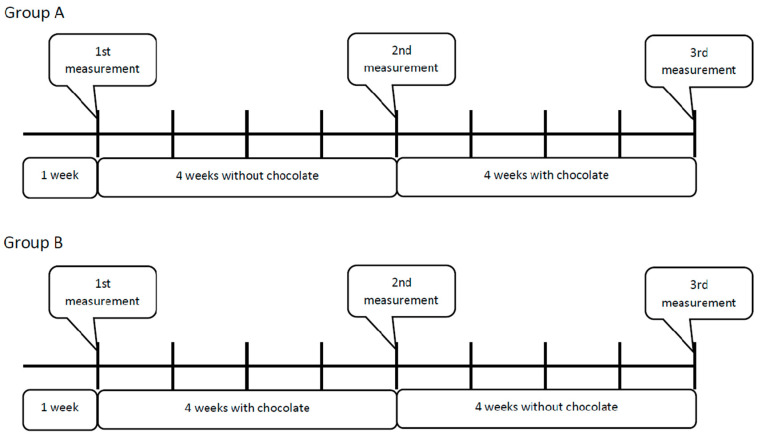
Crossover study design.

The study design is shown in Figure 1.

The study involved 92 participants who were divided into two groups: group A (*n* = 51) and group B (*n* = 41). In the first week, both groups had to follow an anti-inflammatory diet based on the recommendations received on the first visit. After a week, anthropometric parameters were measured and the severity of acne lesions was assessed. Then, group A followed an anti-inflammatory diet for 4 weeks, and group B followed a diet with the addition of 50 g of chocolate with 85% cocoa content per day. After this time, group B started a 4-week anti-inflammatory diet without chocolate, and group A started a 4-week diet with chocolate.

### 2.2. Diet Characteristics

All participants received a ten-day dietary plan with an energy value appropriate to the body’s needs: 1800 kcal, 2000 kcal and 2200 kcal. It was repeated 3 times during the 4-week intervention. The participants’ energy demand was calculated individually for each respondent. For people with a normal body mass index (BMI) of 18.5–24.99 kg/m^2^, basal metabolic rate (BMR) was determined according to the Harris–Benedict formula [17], and for people with BMI over 25 kg/m^2^, BMR was determined according to the Mifflin formula [18]. Total energy requirements were calculated using a physical activity coefficient of 1.4 or 1.6 for people with a normal BMI and a coefficient of 1.4 for overweight people (BMI > 24.9 kg/m^2^). The assumption was that the respondent should maintain a stable body weight throughout the intervention.

An example of the 10-day dietary plan with chocolate for 1800 kcal is presented in the Supplementary Materials (Table S1). The only difference in the diet without chocolate was a recommendation to eat an appropriate amount of dried apricots (40 g) and butter (20 g) instead of chocolate, as an isocaloric equivalent. The subjects received a ten-day dietary plan of 1800 kcal, 2000 kcal or 2200 kcal, divided into 5 meals a day, eaten at regular intervals (every 3–4 h). The anti-inflammatory diet was prepared based on the assumptions of a low-glycemic index diet, limiting products containing simple sugars and products that worsen acne lesions (milk and dairy products, highly processed products and saturated fatty acids). The diet included products that are optimal sources of monounsaturated fatty acids (MUFA) and polyunsaturated fatty acids (PUFA), vitamins and minerals. It also included anti-inflammatory products that are sources of vitamin C, ß-carotene, flavonoids and dietary fiber (including fruit, leafy vegetables, non-starchy vegetables, legumes, tomato juice and lemon), as well as products that are sources of magnesium and vitamin E, such as nuts and seeds. The patients’ diets were enriched with spices such as garlic, onion, dill and parsley leaves. Patients were advised to avoid adding salt to their meals in order to limit sodium intake in their diet. The diets limited pro-inflammatory products, which are sources of saturated fatty acids, such as lard, tropical oils (coconut and palm), pork, fatty meat products and offal, as well as omega-6 PUFA sources like sunflower oil, grape seed oil, corn oil and margarine made from these oils. Salty snacks, sweets, juices and fruit drinks were also limited

The nutrient content and energy percentage of macronutrients in the diets used in the study, which included 50 g/d of chocolate arranged for 3 energy values, are presented in Table 1. The study participants’ compliance with the dietary guidelines was carefully monitored through phone calls after one week of following each diet and via a 24-h dietary interview after two weeks of using each of the diets.

To equalize the energy value and supply of macronutrients in the anti-inflammatory diet, chocolate with 85% cocoa content was replaced by 40 g of dried apricots and 20 g of butter, with a total energy value of 270 kcal. The nutrient content of the chocolate and its isocaloric substitute is presented in Table 2.

### 2.3. Body Composition Analysis

Bioelectrical impedance analysis (BIA) was used for estimating body composition. It is a property-based method of electrical conductance of body tissues. Body composition was assessed in all study participants using the Tanita BC-420 S MA analyzer (TANITA, Tokyo, Japan). The composition analyzer enables quantitative assessment of body composition components using bioelectrical impedance. Metabolic age [years], body water content [%], body weight [kg], BMI [kg/m^2^], body fat content [% and kg] and visceral fat tissue content were also determined. Measurements were carried out after 1 week of application of the anti-inflammatory diet and 4 weeks after the 1st measurement of each dietary intervention.

### 2.4. Statistical Analysis

Continuous variables were presented in tables as mean ± standard deviation (SD). The Mann–Whitney U test was used to compare variables between two study groups at the beginning before they started eating chocolate. The Wilcoxon signed-rank test was used to compare outcomes before and after the applied nutritional intervention (chocolate consumption for 4 weeks). The Mann–Whitney U test was also used to compare changes observed after 4 weeks of chocolate consumption (differences between variables measured before and after chocolate consumption) between the two study groups. The level of statistical significance for all analyses was set at a *p*-value < 0.05. Statistical analysis of the results was performed using STATISTICA v.13 (TIBCO Software Inc., Palo Alto, CA, USA).

## 3. Results

Table 3 shows the comparison between the analyzed anthropometric parameters and the severity of acne lesions between groups A and B before the initiation of the chocolate consumption intervention (the second measurement in group A and the first measurement in group B are shown in Figure 1). The mean age of the participants was similar in groups A and B (24.6 ± 5.5 vs. 25.0 ± 5.1, *p* > 0.05). There were no statistically significant differences in the analyzed parameters between groups.

Table 4 shows a comparison of the studied variables before and after the four-week dietary intervention with chocolate in group A (the second vs. third measurements in group A are shown in Figure 1). In group A, there was a statistically significant reduction in body fat measured in kilograms (from 16.1 ± 8.6 kg before the intervention to 15.8 ± 8.0 kg after 4 weeks on the chocolate diet) and a significant intensification of acne lesions (from 2.5 ± 0.7 to 3.4 ± 0.8 points according to the Investigator’s Static Global Assessment) [16]. No statistically significant changes were found for other parameters (Table 4).

Table 5 shows a comparison of the studied variables before and after the four-week dietary intervention with chocolate in group B (the first vs. second measurements in group B are shown in Figure 1). In group B, most of the tested parameters showed a statistically significant difference between the periods before and after the dietary intervention. A significant intensification of acne lesions (from 2.4 ± 0.7 to 3.5 ± 0.6 points) was observed. A decrease in the average metabolic age was observed, the water content increased, and the content of adipose tissue and body weight of the subjects were also reduced. Only in the case of the content of visceral fat tissue in the body before and after consuming chocolate were no significant differences found (Table 5).

The values of the studied variables obtained before and after 4 weeks of chocolate consumption were compared among all study participants (n = 92). Statistically significant differences were observed in all measured parameters except for visceral fat tissue. The highest statistical significance was noted with reference to the reduction in the amount of adipose tissue (16.4 ± 8.7 to 15.9 ± 8.5 kg; *p* < 0.0001) and the increase in the severity of acne lesions (2.5 ± 0.7 to 3.4 ± 0.7 points; *p* < 0.0001). The results are presented in Table 6.

The differences in the values of the analyzed parameters observed in groups A and B after 4 weeks of following the chocolate diet were also compared. A significantly greater decrease in the BMI value in study participants from group B compared to group A (*p* = 0.0203) was shown, while the difference in body weight of the participants was on the verge of statistical significance (*p* = 0.0542). The observed differences in other parameters were not statistically different between groups (Table 7).

Table 8 shows the change in the severity of acne lesions according to ISGA among subjects from group A (*n* = 51) during the chocolate diet. In 42 out of 51 participants in this group, acne lesions worsened after 4 weeks of consuming chocolate. No change was observed in six subjects. In the case of 35 participants from this group, the severity of acne increased by one point, and in six by two points. Acne lesions decreased in three subjects (two by one point and one by two points).

Similarly, Table 9 shows changes in the severity of acne lesions for group B (*n* = 41). In 37 subjects from group B, the severity of changes was noted, including 30 people who experienced changes that increased by one point according to ISGA and in seven by two points. No changes in acne severity were observed in four subjects.

## 4. Discussion

The aim of this study was to assess the relationship between daily chocolate consumption and the severity of acne lesions. Chocolate is one of the dietary factors suspected of being related to the severity of acne lesions. A review of 53 peer-reviewed articles published between 2009 and 2020 conducted by Dall’Oglio et al. [20] showed that factors contributing to the development of acne include products with high GI/GL, dairy products and chocolate, and the factors that protect against acne are fruits and vegetables as well as unsaturated fatty acids. However, the role played by specific dietary components pertaining to different foods remains an unsolved issue and objective of future research. Different authors have shown that chocolate consumption increases the number of acne lesions [9,21,22]. Research conducted in a group of 25 men with acne lesions showed that daily consumption of 25 g of dark chocolate (99%) for a period of 4 weeks led to an increase in the number of comedones, inflammatory papules and acne score [23]. In turn, a study conducted in a group of 10 men showed that daily consumption of 340 g of chocolate bars for 7 days contributes to an increase in the number of acne outbreaks [21]. It was also found that chocolate consumption increases the presence of Gram-positive microorganisms and epidermal corneocytes in young subjects but not in middle-aged men [24]. However, data on the association between chocolate consumption and the presence of acne are inconsistent, because, for example, one of the studies demonstrated that chocolate consumption increased the production of the anti-inflammatory cytokine, IL-10, induced by *Propionibacterium acne* or *Staphylococcus aureus*, while reducing the release of pro-inflammatory IL-22 [25].

The results observed in this present study indicate that in a significantly larger number of study participants, the presence of chocolate in the diet contributed to the exacerbation of acne by 1 or 2 points, according to the criteria of the 6-point Investigator’s Static Global Assessment (ISGA) scale [16]. In the case of 10 study participants, there was no change in acne lesions as a result of daily consumption of dark chocolate, but in three study participants from group A, the severity of acne lesions decreased. It is probable that these participants did not consume chocolate in the recommended amount and frequency, as indicated by the answers given during the interviews.

It is difficult to explain clearly why chocolate intake is associated with acne lesions. The chocolate with 85% cocoa content used in this study contained cocoa butter, cane sugar, vanilla extract and soy lecithin. Among the ingredients mentioned, cocoa butter and sugar may exacerbate acne symptoms. Slightly more than half of the weight of chocolate is carbohydrates, which can lead to an increase in insulin levels. Insulin and insulin-like growth factor (IGF-1) can promote increased sebum production and the growth of keratinocytes, which leads to clogged pores and the formation of acne lesions [26,27]. Research conducted by Burris et al. [3] shows that a diet with a low GI and GL leads to lower concentrations of IGF-1, which is considered one of the factors in the pathogenesis of acne. Different results were observed in the study conducted by Dougan and Rafikhah [28], which showed that daily consumption of 100 g of white chocolate for 30 days contributed to an increase in the number of non-inflammatory and inflammatory acne lesions. However, consumption of the same amount of dark chocolate was not associated with the exacerbation of acne lesions, which may be due to its low GI.

Dark chocolate has a low GI, so theoretically, it should not exacerbate acne lesions. However, in our study, it was shown that the consumption of 50 g of chocolate had a negative effect on acne lesions. So, it can be argued that GI is not the only parameter that should be taken into account. It is probable that there is also a huge importance of insulin index, as shown by the example of milk. It has been shown that milk (which is a low-GI food) has a negative effect on acne lesions. One of the reasons for this is that milk has a high insulin index because it contains a lot of essential branched-chain amino acids (BCAAs) including leucine, isoleucine and valine, and glutamine. These amino acids in the milk are associated with insulin synthesis and secretion because they communicate with pancreatic β-cells to activate mTORC1. It is worth noting that it is not the content of carbohydrates in milk that has strong insulinotropic effects, but rather the content of insulinotropic amino acids [29]. Therefore, it may be important to consider the BCAA and glutamine content in dark chocolate. Dark chocolate has a low GI, but the content of these amino acids is even higher than in milk (dark chocolate vs. milk 2% fat—leucine: 459 vs. 296 mg/100 g; isoleucine: 262 vs. 190 mg/100 g; valine: 414 vs. 228 mg/100 g; glutamic acid: 1195 vs. 662 mg/100 g) [19]. The intervention in our study was based on the consumption of 50 g of chocolate or food energy equivalent 20 g of butter and 40 g of dried apricots. The content of fats, carbohydrates and protein was similar in these products, but the content of insulinotropic amino acids was about twice as high in the chocolate (Table 2). It is also important to take into account that saturated fatty acids in these products, but also in whole diets, were similar. It is interesting because saturated fatty acids are also considered to be associated with acne lesions [27]. Dark chocolate contains 34.3 g of fat, including 59.5% saturated fatty acids and 39.5% unsaturated fatty acids. Cocoa fat may increase sebum production, but it is moderately comedogenic, which means it tends to clog pores and may cause acne, especially in people with oily or acne-prone skin [2,26,30]. However, it is not clear that they are responsible for the exacerbation of acne lesions after chocolate consumption because, in our study, the content of SFA was similar in the chocolate and the food energy equivalent.

The remaining chocolate ingredients, vanilla extract and soy lecithin, are relatively neutral in terms of their effect on acne. Vanilla extract, despite its antioxidant properties, has no clearly documented effect on acne lesions [31], and soy lecithin is considered an ingredient with a low risk of comedogenicity [32,33].

Most of the studies conducted so far had various limitations. The most common ones include the lack of a control group, small sample size, exclusion of women and short observation period (the severity of acne lesions may appear even after 2 weeks after consuming chocolate). Some studies did not mention important variables such as diet (consumption of sugar, dairy products and the energy value of meals), body weight and BMI, all of which may influence the development of acne. It should also be taken into account that studies are based on responses from participants, and the correctness of data collection also depends on the experience of the researcher. Overall, there is growing evidence in studies since 2011 that chocolate may actually lead to worsening of acne symptoms. However, each study had serious limitations.

This study aimed to eliminate dietary factors that, apart from chocolate, may contribute to the exacerbation of acne lesions. Many researchers indicate that specific dietary patterns may influence the severity of acne symptoms, and appropriate dietary modification may complement drug therapy. The mechanisms by which an anti-inflammatory diet affects acne include the regulation of insulin and IGF-1 levels, which are involved in sebum production and inflammatory processes. Limiting the consumption of products with a high glycemic index and dairy products may reduce the severity of acne symptoms by reducing inflammation and sebum production [2]. Additionally, a diet rich in omega-3 PUFA and antioxidants may have anti-inflammatory effects and improve the condition of the skin. Studies also show that patients following a low glycemic load diet experience a reduction in the severity of acne [2,34]. The study published by Cavicchia et al. [35] and Shivappa et al. [36] is of fundamental importance. They identified dietary ingredients with anti-inflammatory effects based on a review of the medical literature over a period of 60 years describing the impact of food ingredients on the concentration of six known inflammatory markers, interleukins 1β, 4, 6, 10, TNF-α (tumor necrosis factor-alpha) and C-reactive protein. On this basis, dietary ingredients, the consumption of which reduces the levels of inflammation, as assessed by markers in blood serum, were selected. These include vegetables, fruits, herbs, spices, fiber, n-3 PUFA, MUFA, magnesium, flavonoids and carotenoids. They also reduced the severity of acne by limiting sebum production [37,38], whereas SFA, trans fatty acids, high GI carbohydrates, high omega-6/omega-3 PUFA ratio, meat, cheese and alcohol consumption were associated with increased inflammation [39] and worsened skin conditions [27,37]. Therefore, the dietary plan prepared for this study did not include dairy products or industrially processed products. Low-GI products predominated, the content of saturated fatty acids was limited in favor of unsaturated fatty acids, and the diet contained ingredients mainly with anti-inflammatory properties.

It should be noted that the study involved respondents aged 18–45, and the dominant group was women, which distinguishes the study from most other studies conducted with male participants. Acne vulgaris is one of the most common skin diseases. According to the study published by Chen et al. [40], which was based on the analysis from the Global Burden of Disease Study 2019, the age-standardized rates (ASRs) of acne vulgaris increased from 1990 to 2019 both in men and women, but ASRs are higher among women than men. In 2019 the highest prevalence of acne vulgaris was observed in teenagers aged 15–19, but it was also often observed among people over 20 years of age, and even in people over 55 years old. In each age group, the prevalence of acne vulgaris was higher among women than men.

It is justified to conduct further research to determine the impact of chocolate ingredients on the development of acne lesions and to demonstrate whether individual sensitivity to these ingredients may play a role in the development or exacerbation of skin inflammation in acne.

### Limitations

The limitation of this study, similar to many other studies with dietary intervention, was the adherence of study participants to the recommendations. However, we provided a detailed ten-day dietary plan for each participant to follow over the 4 weeks, in addition to the general information about a healthy diet. Moreover, the adherence to the dietary plan was carefully monitored, as described in the Methods section. The ideal situation would be to provide all prepared meals to eat for study participants. Another limitation of this study, also similar to other nutritional studies, is the potential exclusion of the impact of other dietary compounds on the study results. However, to eliminate this problem, the participants received a detailed dietary plan, not only the general recommendations, which excluded other dietary factors known from the literature as having an impact on acne lesion exacerbation.

## 5. Conclusions

The obtained results suggest that daily consumption of 50 g of chocolate with 85% cocoa content, even with an anti-inflammatory diet, may intensify acne lesions in this study group. However, it remains unclear which chocolate components and in what amounts may lead to the exacerbation of acne.

## Figures and Tables

**Table 1:** Nutrient content and energy percentage of macronutrients in each diet with 50 g/d chocolate arranged for 3 energy values.

Dietary Component	1800 kcal (*n* = 10)	2000 kcal (*n* = 10)	2200 kcal (*n* = 10)
	Mean ± SD	Mean ± SD	Mean ± SD
Energy (kcal)	1813.9 ± 13.8	2007.2 ± 15.0	2197.1 ± 11.9
Protein (g)	84.8 ± 2.2	92.6 ± 4	100 ± 4.2
Protein (% energy)	18.7 ± 0.6	18.5 ± 0.9	18.2 ± 0.8
Carbohydrates (g)	236.1 ± 3.7	262 ± 7.1	287.9 ± 5
Carbohydrates (% energy)	48.0 ± 0.8	48.2 ± 1.4	48.8 ± 1.1
Dietary Fiber (g)	36.5 ± 5.7	40.1 ± 5.7	40.1 ± 3.3
Fat (g)	66.8 ± 1.2	74.1 ± 1.3	80.5 ± 2.1
Fat (% energy)	33.2 ± 0.6	33.3 ± 0.8	33.0 ± 0.8
SFA (g)	18.5 ± 1	19.4 ± 1.2	20.2 ± 1.1
SFA (% energy)	9.2 ± 0.5	8.7 ± 0.6	8.3 ± 0.5
MUFA (g)	26.5 ± 2.5	29 ± 2.7	32.1 ± 3.2
MUFA (% energy)	13.2 ± 1.2	13.0 ± 1.3	13.2 ± 1.3
PUFA (g)	16.2 ± 2.2	19.6 ± 2.5	21.4 ± 2.7
PUFA (% energy)	8.1 ± 1.1	8.8 ± 1.1	8.8 ± 1.1
Cholesterol (mg)	264.6 ± 127.7	290.3 ± 157.1	299.6 ± 149.8
Vitamin A—RE (µg)	1731.9 ± 911.5	1748.5 ± 897.3	1770.7 ± 899.8
Retinol RE (µg)	230.7 ± 132.3	246.4 ± 165.5	265.5 ± 167.5
Beta-Carotene Equiv (µg)	9032.1 ± 5425.8	9037.4 ± 5427.2	9064.3 ± 5427.5
Vitamin B1 (mg)	1.3 ± 0.3	1.5 ± 0.3	1.6 ± 0.3
Vitamin B2 (mg)	1.4 ± 0.2	1.5 ± 0.3	1.6 ± 0.3
Vitamin B3 (mg)	25.1 ± 8.2	26.4 ± 8.4	28.2 ± 8.6
Vitamin B6 (mg)	3.0 ± 0.6	3.2 ± 0.6	3.3 ± 0.6
Vitamin B12 (µg)	4.5 ± 3.4	4.6 ± 3.4	4.8 ± 3.6
Vitamin C (mg)	311.5 ± 121.6	312.3 ± 121.5	342.4 ± 121.3
Vitamin D (µg)	7.4 ± 9.0	7.6 ± 9.0	8.0 ± 9.0
Vitamin E (mg)	14.8 ± 2.1	16.1 ± 1.5	17.5 ± 2.5
Folate (µg)	543.3 ± 147.1	572.5 ± 141.5	585.6 ± 141.9
Calcium (mg)	953.0 ± 141.2	933.9 ± 244.0	1026.9 ± 157.3
Iron (mg)	15.5 ± 1.2	16.9 ± 1.8	18 ± 2.3
Magnesium (mg)	578.0 ± 80.0	620.3 ± 74.7	656.6 ± 97.7
Phosphorus (mg)	1533.2 ± 132.4	1687.2 ± 161.8	1804.3 ± 151.8
Potassium (mg)	4136.5 ± 322.0	4377.6 ± 334.4	4567.6 ± 330.4
Sodium (mg)	1458.5 ± 458.3	1580.2 ± 487.6	1699.9 ± 537.0
Zinc (mg)	11.9 ± 1.4	13.1 ± 1.7	13.7 ± 1.9

SFA—saturated fatty acids; MUFA—monounsaturated fatty acids; PUFA—polyunsaturated fatty acids; *n*-number of diets.

**Table 2:** Nutrient content of chocolate and its isocaloric substitute in diet of control group [19].

Nutrient	Chocolate with 85% Cocoa 50 g	Butter 20 g + Dried Apricots 40 g
Energy (kcal)	279.5	270.0
Protein (g)	3.4	2.3
Carbohydrates (g)	28.3	29.0
Total fats (g)	17.2	16.5
SFA (g)	10.7	10.9
Leucine (mg)	229.5	121.2
Isoleucine (mg)	131.0	73.0
Valine (mg)	207.0	90.6
Glutamic acid (mg)	597.5	256.0

SFA—saturated fatty acids.

**Table 3:** Comparison of mean values of age, anthropometric parameters and acne lesions observed before chocolate consumption in group A (*n* = 51) and group B (*n* = 41).

Variables	Group A (*n* = 51)	Group B (*n* = 41)	*p* (Mann–Whitney U Test)
	X ± SD	X ± SD	
Age [years]	24.6 ± 5.5	25.0 ± 5.1	0.5486
Metabolic age [years]	21.4 ± 11.0	21.6 ± 10.7	0.9611
Water content [%]	53.2 ±5.2	52.4 ± 5.7	0.6234
Visceral adipose tissue	2.1 ± 1.9	2.4 ± 1.9	0.3846
Fat tissue [%]	24.8 ± 8.1	25.2 ± 8.4	0.9031
Fat tissue [kg]	16.1 ± 8.6	16.8 ± 9.0	0.7954
BMI [kg/m^2^]	22.3 ± 4.3	22.3 ± 4.1	0.8259
Body weight [kg]	61.8 ± 12.4	62.8 ± 13.0	0.7533
Acne lesions [points]	2.5 ± 0.7	2.4 ± 0.7	0.9688

X ± SD—mean ± standard deviation; BMI—body mass index; *n*-number of participants.

**Table 4:** Comparison of values of anthropometric parameters and acne lesions before and after consuming chocolate in group A (*n* = 51).

Variables	Before Intervention (Before Chocolate)	After Intervention (After Chocolate)	*p* (Wilcoxon Test)
	X ± SD	X ± SD	
Metabolic age [years]	21.4 ± 11.0	20.8 ± 10.2	0.1475
Water content [%]	53.2 ± 5.2	53.5 ± 4.9	0.1010
Visceral adipose tissue	2.1 ± 1.9	2.0 ± 1.8	0.2367
Fat tissue [%]	24.8 ± 8.1	24.6 ± 7.7	0.2220
Fat tissue [kg]	16.1 ± 8.6	15.8 ± 8.0	0.0412
BMI [kg/m^2^]	22.3 ± 4.3	22.2 ± 4.1	0.8214
Body weight [kg]	61.8 ± 12.4	61.5 ± 12.0	0.4720
Acne lesions [points]	2.5 ± 0.7	3.4 ± 0.8	<0.0001

X ± SD—mean ± standard deviation; BMI—body mass index; *n*-number of participants.

**Table 5:** Comparison of values of anthropometric parameters and acne lesions before and after consuming chocolate in group B (*n* = 41).

Variables	Before Intervention (Before Chocolate)	After Intervention (After Chocolate)	*p* (Wilcoxon Test)
	X ± SD	X ± SD	
Metabolic age [years]	21.6 ± 10.7	21.0 ± 10.2	0.0121
Water content [%]	52.4 ± 5.7	53.2 ± 5.4	0.0045
Visceral adipose tissue	2.4 ± 1.9	2.3 ± 1.7	0.1422
Fat tissue [%]	25.2 ± 8.4	24.0 ± 8.8	0.0003
Fat tissue [kg]	16.8 ± 9.0	16.0 ± 9.1	0.0003
BMI [kg/m^2^]	22.3 ± 4.1	22.0 ± 4.1	0.0007
Body weight [kg]	62.8 ± 13.0	62.2 ± 12.8	0.0019
Acne lesions [points]	2.4 ± 0.7	3.5 ± 0.6	<0.0001

X ± SD—mean ± standard deviation; BMI—body mass index; *n*-number of participants.

**Table 6:** Comparison of values of anthropometric parameters and acne lesions before and after consuming chocolate in groups A and B (*n* = 92).

Variables	Before Intervention (Before Chocolate)	After Intervention (After Chocolate)	*p* (Wilcoxon Test)
	X ± SD	X ± SD	
Metabolic age [years]	21.5 ± 10.8	20.9 ± 10.1	0.0095
Water content [%]	52.8 ± 5.4	53.4 ± 5.1	0.0017
Visceral adipose tissue	2.2 ± 1.9	2.1 ± 1.8	0.0692
Fat tissue [%]	24.9 ± 8.2	24.3 ± 8.2	0.0012
Fat tissue [kg]	16.4 ± 8.7	15.9 ± 8.5	<0.0001
BMI [kg/m^2^]	22.3 ± 4.2	22.1 ± 4.1	0.0192
Body weight [kg]	62.3 ± 12.6	61.9 ± 12.3	0.0105
Acne lesions [points]	2.5 ± 0.7	3.4 ± 0.7	<0.0001

X ± SD—mean ± standard deviation; BMI—body mass index; *n*-number of participants.

**Table 7:** Comparison of differences in values of anthropometric parameters and acne lesions observed after 4 weeks of chocolate consumption in group A (*n* = 51) and group B (*n* = 41).

Variables	Group A (*n* = 51)	Group B (*n* = 41)	*p* (Mann–Whitney U Test)
	X ± SD	X ± SD	
Metabolic age [years]	−0.53 ± 2.4	−0.68 ± 1.7	0.4697
Water content [%]	0.33 ± 1.5	0.86 ± 2.1	0.2746
Visceral adipose tissue	−0.08 ± 0.4	−0.10 ± 0.4	1.0000
Fat tissue [%]	−0.16 ± 2.5	−1.14 ± 1.8	0.1355
Fat tissue [kg]	−0.35 ± 1.9	−0.82 ± 1.2	0.2612
BMI [kg/m^2^]	−0.06 ± 0.7	−0.31 ± 0.5	0.0203
Body weight [kg]	−0.25 ± 1.6	−0.60 ± 1.2	0.0542
Acne changes [points]	0.90 ± 0.8	1.07 ± 0.5	0.3178

X ± SD—mean ± standard deviation; BMI—body mass index; *n*-number of participants.

**Table 8:** Difference in severity of acne lesions after 4 weeks of consuming 50 g/day of chocolate in group A (*n* = 51).

Acne Severity Score	Number of Study Participants—Before Intervention (*n* = 51)	Changes after 4-Week Dietary Intervention (*n* = 51)					
		No Change	+1 Point	+2 Points	+3 Points	−1 Point	−2 Points
0	0	0	0	0	0	0	0
1	5	0	2	2	1	0	0
2	19	2	14	3	0	0	0
3	25	4	18	1	0	1	1
4	2	0	1	0	0	1	0
5	0	0	0	0	0	0	0

**Table 9:** Difference in severity of acne lesions after 4 weeks of consuming 50 g/day of chocolate in group B (*n* = 41).

Acne Severity Score	Number of Study Participants—Before Intervention (*n* = 41)	Changes after 4-Week Dietary Intervention (*n* = 41)					
		No Change	+1 Point	+2 Points	+3 Points	−1 Point	−2 Points
0	0	0	0	0	0	0	0
1	5	0	1	4	0	0	0
2	13	0	10	3	0	0	0
3	23	4	19	0	0	0	0
4	0	0	0	0	0	0	0
5	0	0	0	0	0	0	0

## Data Availability

The original contributions presented in the study are included in the article/Supplementary Materials, further inquiries can be directed to the corresponding author.

## Supplementary Materials

The following supporting information can be downloaded at https://www.mdpi.com/article/10.3390/foods13131993/s1, Table S1: Ten-day dietary plan prepared for 1800 kcal, divided into meals, which was given to the participants (version with chocolate; the second group received the same diet but with 20 g butter and 40 g dried apricots instead of chocolate as an caloric equivalent).
