# Supplementary material for: The Relationship between Chocolate Consumption and the Severity of Acne Lesions−A Crossover Study
Source: Foods. 2024 Jun 24;13(13):1993. doi: 10.3390/foods13131993 (PMC11241376; doi:10.3390/foods13131993)
Supplement: Supplementary file 1 [file foods-13-01993-s001.zip › foods-3046452-supplementary.pdf]

Table S1. Ten-day dietary plan prepared for 1,800 kcal, divided into meals, which was given to the participants (version with chocolate; the second group received the same diet but with 20 g butter and 40 g dried apricots instead of chocolate as an caloric equivalent).

Day 1

Calcium-rich mineral water	1.5 L

Breakfast

Whole-grain rye bread	60 g
Lettuce	10 g
Tuna in its own juice	90 g
Pickled cucumber	90 g
Onion	20 g
Red pepper	50 g

Mix the tuna with olive oil. Season, chop the cucumber, finely chop the onion and pepper. Mix all the ingredients. Arrange the lettuce onto bread slices and apply the paste.

Dark chocolate	25 g

Second breakfast

Spinach	50 g
Orange	110 g
Lemon	60 g
Mineral water	120 g
Flax seeds	5 g

Blend all the ingredients.

Dark chocolate	25 g

Lunch

Durum wheat pasta	60 g
Chicken breast meat without skin	60 g
Tomato	200 g
Lettuce	20 g
Olive oil	10 g
Parsley	15 g
Walnuts	15 g

Boil the pasta. Stew the meat cut into cubes and seasoned with your favorite herbs in a frying pan without fat. Cut the tomato, chop the parsley, lettuce and nuts. Mix.
(the dish can be prepared for dinner and divided, measure the pasta separately for meals)

Afternoon snack

Oatmeal	30 g
Apple	150 g
Almonds	10 g

Cut the apple into pieces, sprinkle with oatmeal and almonds.

Dinner

Durum wheat pasta	50 g
Chicken breast meat without skin	60 g
Tomato	200 g
Lettuce	20 g
Olive oil	10 g
Parsley	15 g
Walnuts	5 g

The dish should already be prepared.

Day 2

Calcium-rich mineral water	1.5 L

Breakfast

Eggs, 2 pcs.	112 g
Chives	10 g
Tomato	120 g
Whole-grain rye bread	60 g

Lightly heat the pan, add the eggs and chopped chives. Curdle the eggs in a pan. Cut the tomatoes into pieces.

Dark chocolate	25 g

Second Breakfast

Cucumber	90 g
Celery	45 g
Red pepper	70 g
Carrot	90 g
Whole-grain rye bread	30 g

Cut the vegetables into sticks and toast the bread any way you like.

Dark chocolate	25 g

Lunch

Turkey breast meat without skin	100 g
Cold pressed rapeseed oil	10 g
Lemon juice	6 g
Parsley	10 g
Cucumber	90 g
Tomato	100 g
Barley groats	80 g
Homemade vegetable broth	125 g

Boil barley groats, add broth. You can also add some water. Cut vegetables into cubes, and chop the parsley, mix with groats and add spices for better flavor. Grill the turkey in the pan or electric pan, or, stem or stew in a pan without fat. Prepare the meal for a dinner, divide according to the grammage, turkey and groats separately.

Afternoon snack

Pear	130 g
Walnuts	20 g

Wash the fruit and eat it with walnuts.

Dinner

Turkey breast meat without skin	75 g
Cold pressed rapeseed oil	10 g
Lemon juice	6 g
Parsley	10 g
Cucumber	90 g
Tomato	100 g
Barley groats	50 g
Homemade vegetable broth	125 g

The dish should already be prepared.

Day 3

Calcium-rich mineral water	1.5 L

Breakfast

Egg, 1 pc.	56 g
Chicken breast, tenderloin	30 g
Tomato	60 g
Red pepper	50 g
Whole-grain rye bread	60 g
Lettuce	20 g

Boil the egg and slice it. Cut the red pepper into stripes and slice the tomatoes. Spread butter on bread, make sandwiches.

Dark chocolate	25 g

Second Breakfast

Spinach	25 g
Walnuts	5 g
Grapefruit	100 g
Soya milk	150 g

Mix all the ingredients in the blender. You can add some water for the preferred consistency.

Dark chocolate	25 g

Lunch

Basmati rice	50 g
Avocado	35 g
Carrot	45 g
Red pepper	70 g
Chives	10 g
Cucumber	90 g
Oven-roasted salmon	100 g

Roast the salmon in the oven. Boil the rice. Prepare vegetables in the form of salad. Prepare the dish also for dinner, divide ingredients according to the grammage.

Afternoon snack

Onion	25 g
Tomato	120 g
Celery	40 g
Lettuce	50 g
Orange	200 g
Whole-grain rye bread	30 g
Olive oil	5 g
Mustard	10 g

Use mentioned above ingredients to make a salad, add dressing made from olive oil and mustard.

Dinner

Basmati rice	50 g
Avocado	35 g
Carrot	45 g

Red pepper	70 g
Chives	10 g
Cucumber	90 g
Oven-roasted salmon	50 g

The dish should already be ready.

Day 4

Calcium-rich mineral water	1.5 L

Breakfast

Egg, 1 pc.	56 g
Whole-grain rye bread	60 g
Spinach	25 g
Tomato	120 g
Red pepper	30 g
Onion	20 g

Prepare toasts from your bread slices. Put spinach on top of them. Heat the pan. Beat the egg with the fork and pour into the pan. When they curdle, divide the omelette into halves, fold them into squares to fit the toasts. Put the omelette on the toasts. Cut the tomato and pepper into pieces and eat with toasts.

Dark chocolate	25 g

Second Breakfast

Lettuce	50 g
Pear	130 g
Olive oil	10 g
Walnuts	10 g
Dried apricots	20 g

Slice the pear, remove the seeds. Add walnuts and apricots, pour olive oil over the salad.
Prepare the dish also for the Afternoon snack.

Dark chocolate	25 g

Lunch

Buckwheat groats	100 g
Grilled cod fillet	100 g
Parsley root	75 g
Zucchini	150 g
Cold-pressed rapeseed oil	5 g
Dill	5 g

Season the cod for a better flavor, sprinkle lemon juice over it. Boil the groats in water. Cut vegetables into slices or pieces, season them and grill with the fish in 180°C until they become soft. You can sprinkle the groats with the oil from fish and vegetables

Prepare the dish also for dinner. Divide the groats according to the grammage.

Afternoon snack

Lettuce	50 g
Pear	130 g
Olive oil	10 g
Dried apricots	20 g

The dish should already be prepared.

Dinner

Buckwheat groats	50 g
Grilled cod fillet	50 g
Parsley root	75 g
Zucchinni	150 g
Cold-pressed rapeseed oil	5 g
Dill	5 g

The dish should already be prepared.

Day 5

Calcium-rich mineral water	1.5 L

Breakfast

Whole-grain rye bread	90 g
Tuna in its own sauce	50 g
Tomato	60 g
Lettuce	20 g
Olive oil	5 g

Put the ingredients onto your sandwich.

Dark chocolate	25 g

Second Breakfast

Walnuts	20 g
Dark chocolate	25 g

Lunch

Barley groats	50 g
Red lentils, dry seeds	25 g
Turkey drumstick meat without skin	100 g
Onion	20 g
Red pepper	50 g
Broccoli	150 g
Olive oil	5 g

Cook the groats. Cut the meat into strips, stew in a pan, add lentils and chopped pepper. Mix everything together and season with herbs and spices as desired. Prepare the dish also for dinner. Separate the groats and turkey according to weight.

Afternoon snack

Frozen raspberries	200 g
Walnuts	10 g
Soya yogurt	150 g

Add raspberries and walnuts to the yogurt.

Dinner

Barley groats	30 g
Red lentils, dry seeds	25 g
Turkey drumstick meat without skin	50 g
Onion	20 g
Red pepper	50 g
Broccoli	150 g
Olive oil	5 g

The dish should already be prepared.

Day 6

Calcium-rich mineral water	1.5 L

Breakfast

Whole-grain rye bread	90 g
Lettuce	20 g
Tomato	120 g
Turkey ham	40 g

Prepare sandwiches.

Dark chocolate	25 g

Second Breakfast

Walnuts	20 g
Dark chocolate	25 g

Lunch

Durum wheat pasta	80 g
Turkey breast meat without skin	100 g
Olive oil	10 g
Garlic	3 g
Onion	25 g
Tomato pulp	200 g

Heat the olive oil in a pan, add the chopped onion and pressed garlic. Add salt and simmer over low heat until the onion becomes glassy. Add the chopped meat and fry until it is no longer raw. Add spices and pepper. When the meat is cooked, you can add tomato pulp. Stew everything together covered, stirring from time to time. If the sauce is too thin, simmer without a lid until some of the water evaporates.
Prepare the dish for dinner, add pasta according to weight.

Afternoon snack

Oat flakes	30 g
Soya milk	100 g
Pumpkin seeds	10 g
Frozen strawberries	200 g

Mix the flakes with milk, then add pumpkin seeds and previously chopped strawberries.

Dinner

Durum wheat pasta	30 g
Turkey breast meat without skin	100 g
Olive oil	10 g
Garlic	3 g
Onion	25 g
Tomato pulp	200 g

The dish should already be prepared.

Day 7

Calcium-rich mineral water	1.5 L

Breakfast

Whole-grain rye bread	90 g
Tomato	60 g
Cucumber	90 g
Chicken breast tenderloin	40 g

Prepare sandwiches.

Dark chocolate	25 g

Second Breakfast

Soya yogurt	150 g
Sesame seeds	15 g
Dark chocolate	25 g

Add sesame seeds and chocolate to the yogurt.

Lunch

Buckwheat groats	50 g
Sauerkraut	80 g
Carrot	30 g
Olive oil	10 g
Chicken breast meat without skin	150 g
Onion	40 g
Red pepper	40 g

Cook the groats. Grease the pan with some olive oil. Season the meat with pepper, marjoram and other spices as desired, add the rest of the olive oil and other ingredients. Stew everything. Eat with cooked groats and sauerkraut.

Afternoon snack

Whole-grain rye bread	60 g
Hummus	60 g
Chives	5 g
Radish	120 g

Prepare sandwiches.

Dinner

Red pepper	100 g
Tomato	120 g
Onion	40 g
Garlic	3 g
White beans, dry seeds	20 g
Tomato juice	80 g
Cold-pressed rapeseed oil	15 g
Whole-grain rye bread	30 g

Cook the beans and then stew everything until the desired consistency is achieved.

Day 8

Calcium-rich mineral water	1.5 L

Breakfast

Whole-grain rye bread	60 g
Turkey loin	40 g
Tomato	60 g
Radish	60 g

Prepare sandwiches.

Dark chocolate	25 g

Second Breakfast

Dark chocolate	25 g
Frozen raspberries	150 g
Flax seeds	5 g
Mineral water	150 g
Banana	60 g
Almonds	10 g

Mix all the ingredients in the blender.

Lunch

Chicken breast meat without skin	110 g
Whole-grain rye bread	30 g
Olive oil	10 g
Walnuts	10 g
Pear	40 g
Lettuce	40 g
Durum wheat pasta	50 g

Cook the pasta and grill the meat. Cut the pear into slices, place the slices on a grill pan and heat until they soften and form strips. Mix the salad ingredients and place pear slices on top.

Afternoon snack

Whole-grain rye bread	60 g
Olive oil	5 g
Tuna in its own juice	80 g
Tomato	120 g
Dill	15 g

Mix the tuna with olive and dill. Put all the ingredients on the bread.

Dinner

Egg, 1 pc.	56 g
Cucumber	54 g
Tomato	60 g
Whole-grain rye bread	60 g
Olive oil	5 g
Champignons	50 g

Make scrambled eggs in heated olive oil with mushrooms, chop the vegetables. Serve scrambled eggs with bread, tomato and cucumber.

Day 9

Calcium-rich mineral water	1.5 L

Breakfast

Whole-grain rye bread	60 g
Tomato	120 g
Egg, 1 pc.	56 g
Radish	60 g

Boil the egg hard. Put the ingredients on the bread

Dark chocolate	25 g

Second Breakfast

Dark chocolate	25 g
Soya yogurt	150 g
Walnuts	10 g

Add the ingredients to the yogurt.

Lunch

Turkey breast meat without skin	150 g
Olive oil	10 g
Garlic	3 g
Canned corn	20 g
Spinach	100 g
Durum wheat pasta	75 g

Cook the pasta in water. Cut the meat into cubes, pour half of the olive oil and mix. Chop the garlic or press it. Put the meat in olive oil into the pan and fry on both sides for about 5 minutes. Put the finished meat into a separate bowl. Pour the rest of the olive oil into the same pan, add the garlic, spinach and simmer over low heat, covered. Add 1/5 cup of water and mix. Set a low flame, add the meat and corn. Taste and season as desired. Add the pasta and mix everything thoroughly.

Afternoon snack

Flax seeds	10 g
Orange	200 g
Spinach	100 g
Pineapple	120 g
Mineral water	120 g

Mix all the ingredients in the blender

Dinner

Avocado	35 g
Marinated green olives	15 g
Tomato	100 g
Whole-grain rye bread	60 g
Lemon juice	6 g
Smoked mackerel	50 g

Prepare the paste: mash the mackerel and avocado with a fork, season with lemon juice. Add olives. Spread the paste and tomato onto the bread.

Day 10

Calcium-rich mineral water	1.5 L

Breakfast

Egg, 1 pc.	56 g
Red pepper	30 g
Onion	50 g
Parsley	12 g
Whole-grain rye bread	60 g

Chop the onion, pepper and fry. Add chopped parsley to the pan and season to taste. Beat the egg with two tablespoons of water in a bowl and season. Fry eggs into an omelette. Chop the avocado and mix with the fried vegetables. Fill the omelette with the vegetable mixture and fold it in half.

Dark chocolate	25 g

Second Breakfast

Soya milk	150 g
Frozen raspberries	100 g
Oat flakes	20 g
Dark chocolate	25 g

Mix all the ingredients in the blender.

Lunch

Fresh rainbow trout	200 g
Barley groats	50 g
Tomato	40 g
Cucumber	40 g
Parsley	6 g
Lemon juice	9 g
Olive oil	5 g
Red pepper	40 g

Sprinkle the trout with lemon and season. Wrap it in foil and stew, and when it is ready, unwrap the foil and bake the fish on the outside side. Bake at 160-180°C. You can also use an electric grill or grill pan.
Cook the groats, cut the vegetables into cubes and the parsley into small pieces. Mix with groats, olive oil and season.

Afternoon snack

Frozen raspberries	50 g
Soya milk	100 g
Oat flakes	30 g

Chop the fruit and add it to the milk together with the oat flakes.

Dinner

Whole-grain rye bread	90 g
Radish	90 g
Cucumber	40 g
Tofu	60 g
Almond milk	50 g
Onion	20 g

Carrot	45 g
Avocado	35 g

Place the tofu in a bowl. Add almond milk and blend in the form of paste. Cut the vegetables into small cubes and add them to the tofu. Season and mix everything.
